# Effects of high-flux hemodialysis and hemodiafiltration on the mortality of patients with end-stage kidney disease: a meta-analysis

**DOI:** 10.1080/0886022X.2022.2147436

**Published:** 2023-07-10

**Authors:** Sihui Ma, Nan Pu, Juan Ma

**Affiliations:** a Department of Nephrology Center, Beijing Luhe Hospital, Capital Medical University, Beijing, China; b Department of Clinical Laboratory Medicine, Beijing Shijitan Hospital, Capital Medical University, Beijing, China

**Keywords:** High-flux hemodialysis, hemodiafiltration, end-stage kidney disease, survival outcome

## Abstract

**Background:**

High-flux hemodialysis (HFHD) is widely used in hemodialysis centers and is the mode of hemodialysis actively recommended by the guidelines. Additionally, hemodiafiltration (HDF) is widely used in clinical practice. However, there are some inconsistencies in the results of studies on the effects of HDF and HFHD, which has caused controversy regarding which of these two dialysis modalities to select.

**Objective:**

To explore the effect of HFHD and HDF on the survival of patients with end-stage kidney disease (ESKD).

**Methods:**

A systematic search of the PubMed, EMBASE, Cochrane Library, CNKI, Wanfang, and VIP databases was conducted, focusing on cohort studies and randomized controlled trials on hemodialysis in patients with ESKD using HFHD or HDF. A meta-analysis of all-cause mortality and cardiovascular mortality was conducted using Review Manager 5.3 software, and fixed and random effect models were applied according to the heterogeneity results.

**Results:**

A total of 13 studies, including six cohort studies and seven randomized controlled trials, were included in the final analysis. The results revealed that HFHD had no statistically significant effect on the all-cause mortality (odds ratio (OR): 1.16, 95% confidence interval (CI): 0.86, 1.57) or cardiovascular mortality (OR: 0.86, 95% CI: 0.64, 1.15) of patients with ESKD. However, compared with HDF, HFHD reduced the infection mortality rate (OR: 0.50, 95% CI: 0.33, 0.77).

**Conclusions:**

Compared with HDF, HFHD has no obvious benefits for all-cause mortality or cardiovascular mortality in patients with ESKD, but reduced risk of infection-related death.

## Introduction

Chronic kidney disease (CKD) is a serious public health problem. According to a survey, the prevalence rate was 9.1% in 2017, affecting 690 million people [1]. End-stage kidney disease (ESKD) is a disease that seriously affects the quality of life and life expectancy of patients and has a global prevalence rate of approximately 0.07%. There are approximately 132 million patients with CKD in China, and approximately 2% of patients develop ESKD every year. However, the 5-year survival rate of patients with ESKD is only 39% [2,3]. Moreover, the medical costs associated with ESKD are still increasing [4,5]. Therefore, ESKD harms the quality of life of patients and is a great challenge for health insurance.

Currently, kidney transplantation, peritoneal dialysis, and hemodialysis are three common treatments for patients with ESKD. Kidney transplantation is limited in the application stage due to the lack of appropriate kidney sources, patients’ age, and severe illness. Hemodialysis is not only suitable for most patients with nephropath but also can be used as a transitional treatment for kidney transplantation. Currently, approximately 2 million patients with ESKD worldwide are treated with hemodialysis to sustain their lives [6]. With the development and improvement of hemodialysis technology, four main hemodialysis technologies are available: hemofiltration (HF), hemodiafiltration (HDF), low-flux hemodialysis (LFHD), and high-flux hemodialysis (HFHD).

Compared with LFHD, HDF and HFHD not only effectively remove small molecule toxins but also have obvious advantages in removing macromolecules [7]. In Europe, HFHD is widely used in hemodialysis centers and is the hemodialysis mode actively recommended by the guidelines. In addition, HDF is widely used in clinical practice due to its ability to decrease the mortality rate [8–10]. However, there are some inconsistencies in the results of studies on the effects of HDF and HFHD, which has caused controversy regarding which of these two dialysis modalities to select. Some studies have shown that HDF has no statistical significance in reducing all-cause mortality [11,12]. Others suggest that HDF can improve the survival benefits of patients [13–15]. Therefore, this study systematically compares the application effect of HDF and HFHD on patients with ESKD and aims to provide a clinical practice basis for the selection of hemodialysis mode in patients with ESKD.

## Materials and methods

### Retrieval strategy

In line with the Preferred Reporting Items for Systematic Reviews and Meta-Analyses guidance manual, three English databases, PubMed, EMBASE, and Cochrane Library, and three Chinese databases, CNKI, Wanfang, and VIP, were systematically searched. The search period was up to 31 July 2022. English database retrieval strategies included the following keywords: ‘end-stage kidney disease’, ‘hemodiafiltration’, and ‘high-flux hemodialysis’. Chinese database retrieval strategies include the same Chinese keywords (see the supplemental file for detailed retrieval strategy).

### Inclusion and exclusion criteria

The inclusion criteria are as follows: (1) study participants aged over 18 years and diagnosed with ESKD; (2) when using HFHD and HDF, the other treatment measures are as follows: hemodialysis frequency is 2–3 times a week, each dialysis time is ≥4 h and maintenance hemodialysis time is ≥3 months; (3) HFHD and HDF are mutually compared; (4) main study outcomes are mortality and cardiovascular mortality; (5) the study type is a cohort study or randomized controlled trial; and (6) follow-up time is ≥1 year.

The exclusion criteria are as follows: (1) participants suffering from a tumor or other life-threatening complications or combined use of other kidney therapies; (2) studies on non-population; (3) conference papers, case reports, systematic reviews, and other study types; (4) insufficient outcome information and data analysis; and (5) literature research on repeated reports.

### Literature screening and data extraction

Two reviewers (M.S.H., P.N.) independently screened the literature according to the inclusion and exclusion criteria. When the two opinions were inconsistent, a third reviewer (M.J.) was consulted to reach a unified opinion. After the literature screening, the data were extracted by two reviewers (M.S.H., P.N.), which included the literature information, demographic characteristics of the participants, hemodialysis technology, related outcome indicators, study types, and other information.

### Quality assessment

The Newcastle–Ottawa Scale (NOS) was used to evaluate the quality of the cohort studies. The scale was evaluated based on the following eight items: representativeness of cohort, choice of the non-exposed cohort, comparability of the cohort, adequacy of outcome evaluation, adequacy of follow-up time, and completeness of follow-up. The full score was 9. The total score was 7 and above for high-quality literature and 5 and below for low-quality articles. The Cochrane Collaboration’s tool for assessing the risk of bias was used to evaluate the quality of the randomized controlled trials. The randomized allocation method, allocation concealment, blinding method, integrity of the results data, selective reporting of research results, and other sources of bias were evaluated.

### Statistical analysis

Review Manager 5.3 software was used for the statistical analysis. The effect of the count data was represented by the odds ratio (OR), and the 95% confidence interval (CI) was used to estimate the interval range of the effect. The *I*^2^ test was used to determine heterogeneity. If *I*^2^<50% or *p* > 0.1, the included literature was homogeneous. The fixed effect model (Mantel–Haenszel) was used for the analysis. If *I*^2^>50% or *p* ≤ 0.1, the included studies were not homogeneous. Moreover, the random effect model (DerSimonian–Laird) was used for the analysis. If the heterogeneity was large, a sensitivity analysis was used to explore the source of heterogeneity. A funnel plot was used to evaluate the bias of the literature. *p* < 0.05 indicated that the difference was statistically significant.

## Results

### Characteristics of the included literature

**Figure 1.**
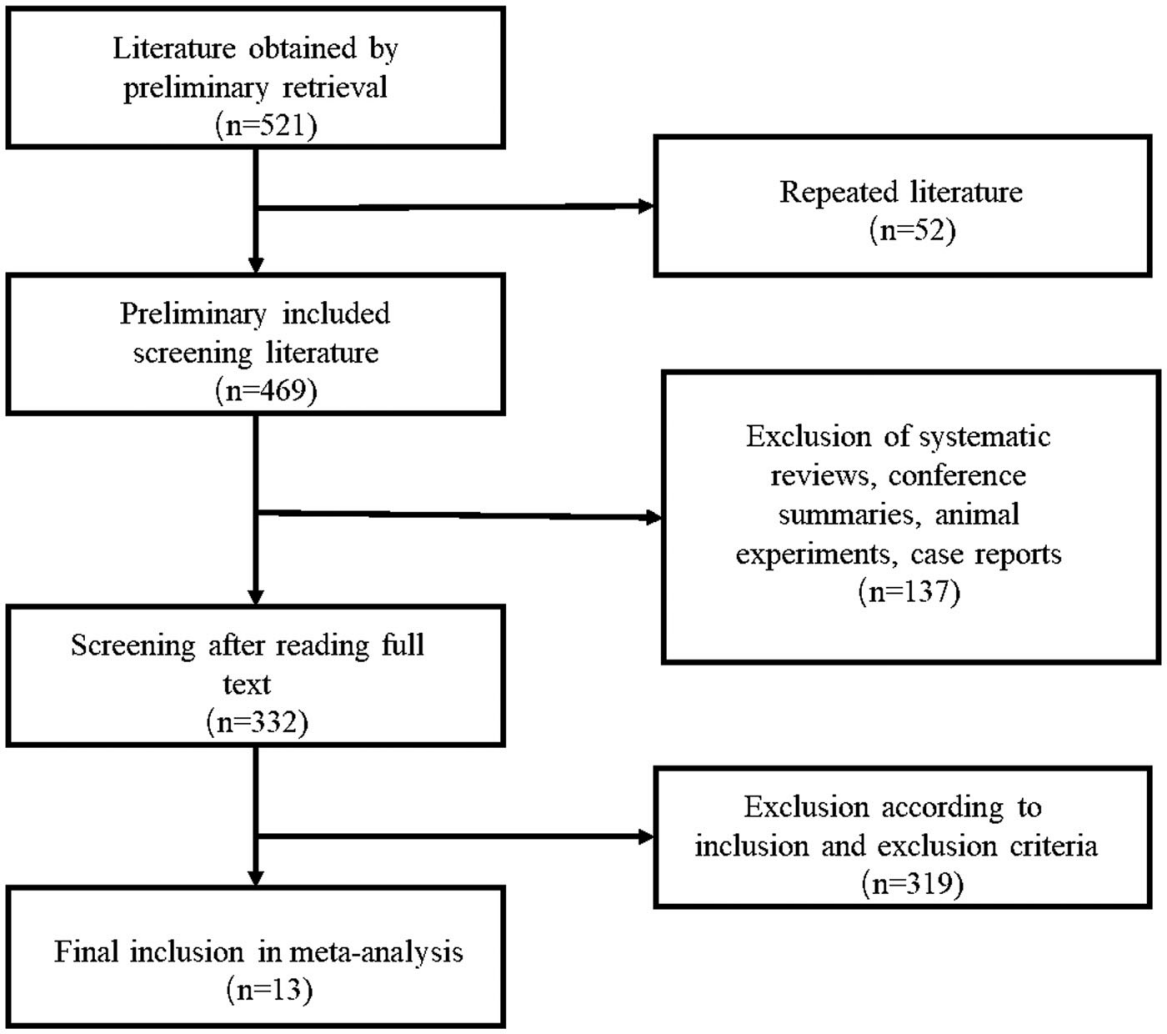
Flowchart of literature screening.

A total of 521 studies were retrieved according to the retrieval strategy, and 13 studies were included in the meta-analysis after literature screening [11–14,16–24]. The flowchart of the literature screening is presented in Figure 1. Table 1 shows the basic characteristics of the included studies. There were two prospective cohort studies, four retrospective cohort studies and seven randomized controlled trials. Nine studies were conducted in European countries, and only one was conducted in China. Moreover, 10 studies used online HDF technology. A total of 7796 participants were enrolled in 13 studies, including 4156 in the HFHD group and 3640 in the HDF group. The longest follow-up time was 6.6 years.

**Table 1.** General characteristics of the included literature.

Study	Location	Hemodialysis mode	Sample size	Study design	Follow-up (year)	Age	Female (%)	NOS score
Canaud et al. [17]	Europe	HFHD	546	Prospective cohort study	3	58.5	41.9	7
		OLHDF	253			62.5	39.5	
Schiffl [19]	Germany	HFHD	38	RCT	2	59 ± 10	47.4	–
		OLHDF	38			63 ± 9	42.1	
Vilar et al. [16]	UK	HFHD	626	Retrospective cohort study	5	64.2 ± 13.9	37.4	8
		OLHDF	232			53.1 ± 15.7	16.8	
Locatelli et al. [24]	Italy	HFHD	40	RCT	2.2	54.6–74.3	45	–
		HDF	70			58.1–70.8	38.6	
Grooteman et al. [11]	Netherlands, Canada, Norway	OLHDF	358	RCT	6.6	64.1 ± 14.0	40	–
		HFHD	356			64.0 ± 13.4	35	
Maduell et al. [13]	Spain	OLHDF	456	RCT	3	64.5 ± 14.4	30.5	–
		HFHD	450			66.3 ± 14.3	35.8	
Ok et al. [12]	Turkey	HFHD	391	RCT	2	56.5 ± 14.9	41.9	–
		OLHDF	391			56.4 ± 13.0	40.4	
Imamović et al. [20]	Balkan region	HFHD	151	Retrospective cohort study	5	65 ± 12	37.7	8
		OLHDF	291			65 ± 15	36.4	
Canaud et al. [14]	Europe	HFHD	795	Retrospective cohort study	5	65 ± 15	34.0	8
		OLHDF	795			65 ± 14	36.0	
Djuric et al. [21]	Serbia	HFHD	32	Prospective cohort study	3	57.1 ± 9.6	71.9	8
		HDF	26			57.4 ± 10.3	53.8	
Maduell et al. [18]	Spain	OLHDF	506	Retrospective cohort study	6	67.75 ± 13.32	35.6	7
		HFHD	506			67.68 ± 13.47	33.8	
Morena et al. [23]	France	HFHD	191	RCT	2	76.11 ± 6.68	39.8	–
		OLHDF	190			76.35 ± 6.13	40.0	
Lei [22]	China	HDF	34	RCT	6	25–75	NA	–
		HFHD	34				NA	

HFHD: high-flux hemodialysis; HDF: hemodiafiltration; OLHDF: online hemodiafiltration; NA: not described.

### Literature quality assessment

**Figure 2.**
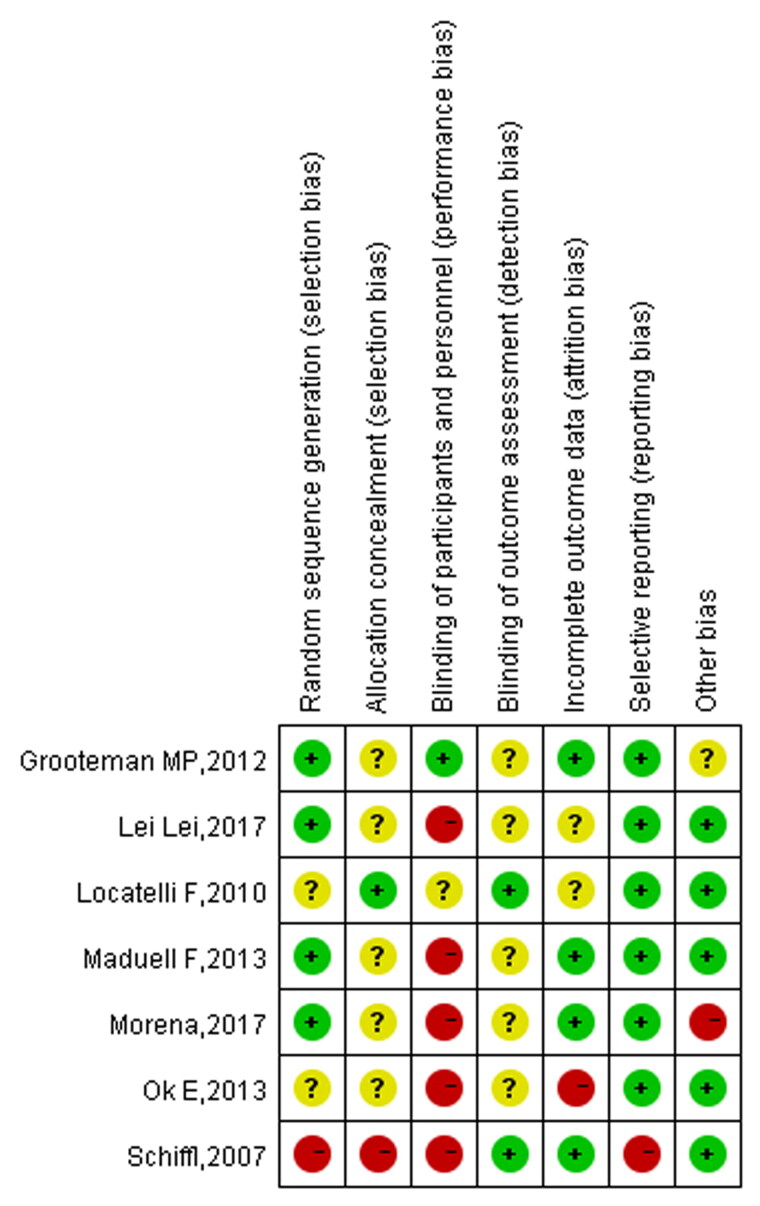
Summary of risk of bias in RCTs.

**Figure 3.**
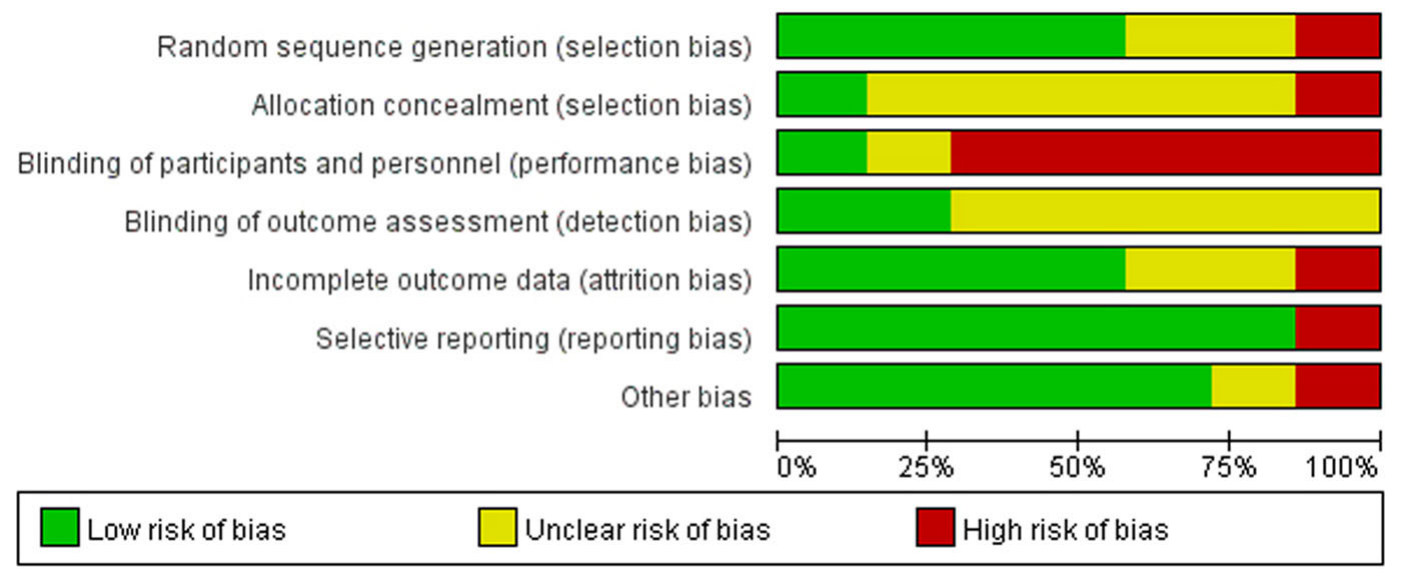
Bias risk diagram.

The average NOS score in the cohort studies was 7.7, as two studies scored 7 and four studies scored 8. The quality of the cohort studies included in the analysis was high, as shown in Table 1. The quality assessment results of the randomized controlled trials show that the quality of the seven studies was relatively low, with the risk mainly coming from the implementation and distribution of blind methods. The risk of reporting bias and other biases was low, as illustrated in Figures 2 and 3.

### Effects of HFHD and HDF on the all-cause mortality of patients

**Figure 4.**
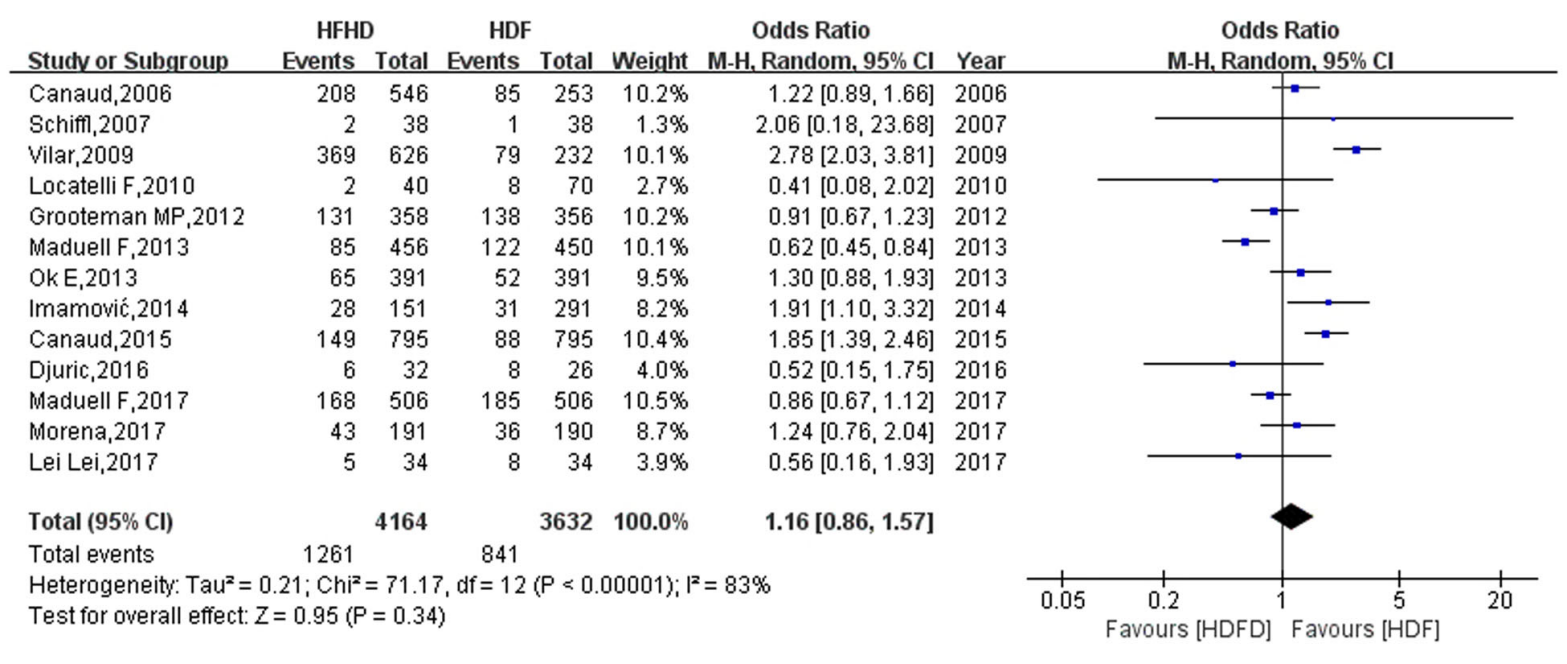
Comparison results of all-cause mortality between HFHD and HDF.

All 13 studies reported the all-cause mortality of patients after HFHD and HDF. The heterogeneity test results were *I*^2^=83% and *p* < 0.00001, suggesting that the heterogeneity of the included studies was large. Moreover, a random effect model was used for the meta-analysis. The comparison of all-cause mortality between HFHD and HDF (Figure 4) revealed that there were 841 deaths in the HDF group and 1261 deaths in the HFHD group. All-cause mortality in the HFHD group was higher than that in the HDF group, and the effector OR was 1.16 (95% CI: 0.86, 1.57). However, this difference was not statistically significant (*p* = 0.34).

### Effect of HFHD and HDF on cardiovascular mortality in patients

**Figure 5.**
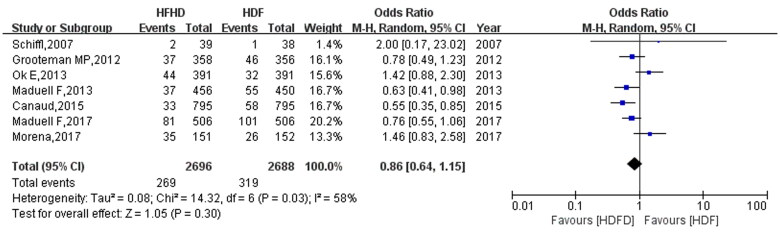
Comparison of cardiovascular mortality between HFHD and HDF.

Seven studies reported death from cardiovascular disease after HFHD and HDF. The heterogeneity test showed that *I*^2^=58% and *p* = 0.03, indicating that the heterogeneity between the studies was relatively high, and the random effect model was used to estimate the effect. The comparison results of cardiovascular disease mortality between HFHD and HDF (Figure 5) revealed no significant difference in the effect of HFHD and HDF on cardiovascular disease mortality in patients with ESKD (OR: 0.86, 95% CI: 0.64, 1.15, *p*= 0.30). After excluding one study, the heterogeneity was reduced by 17%, and a statistically significant difference was identified (OR: 0.75, 95% CI: 0.62, 0.91).

### Effect of HFHD and HDF on infection mortality

**Figure 6.**
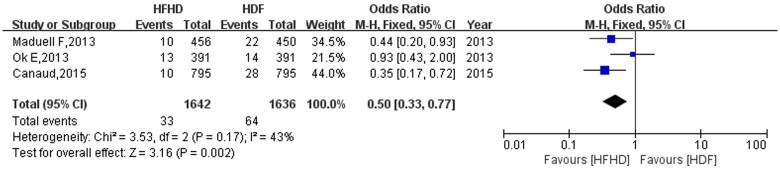
Comparison of infection mortality between HFHD and HDF.

Three studies reported the results of infection mortality. There was no obvious heterogeneity in the included studies (*I*^2^=43%, *p* = 0.17), and the fixed effect model was used. The meta-analysis results showed that HFHD reduced infection mortality. The combined effect was 0.50 (95% CI: 0.33, 0.77, *p* = 0.002) (Figure 6).

### Subgroup analysis of all-cause mortality and cardiovascular mortality

**Figure 7.**
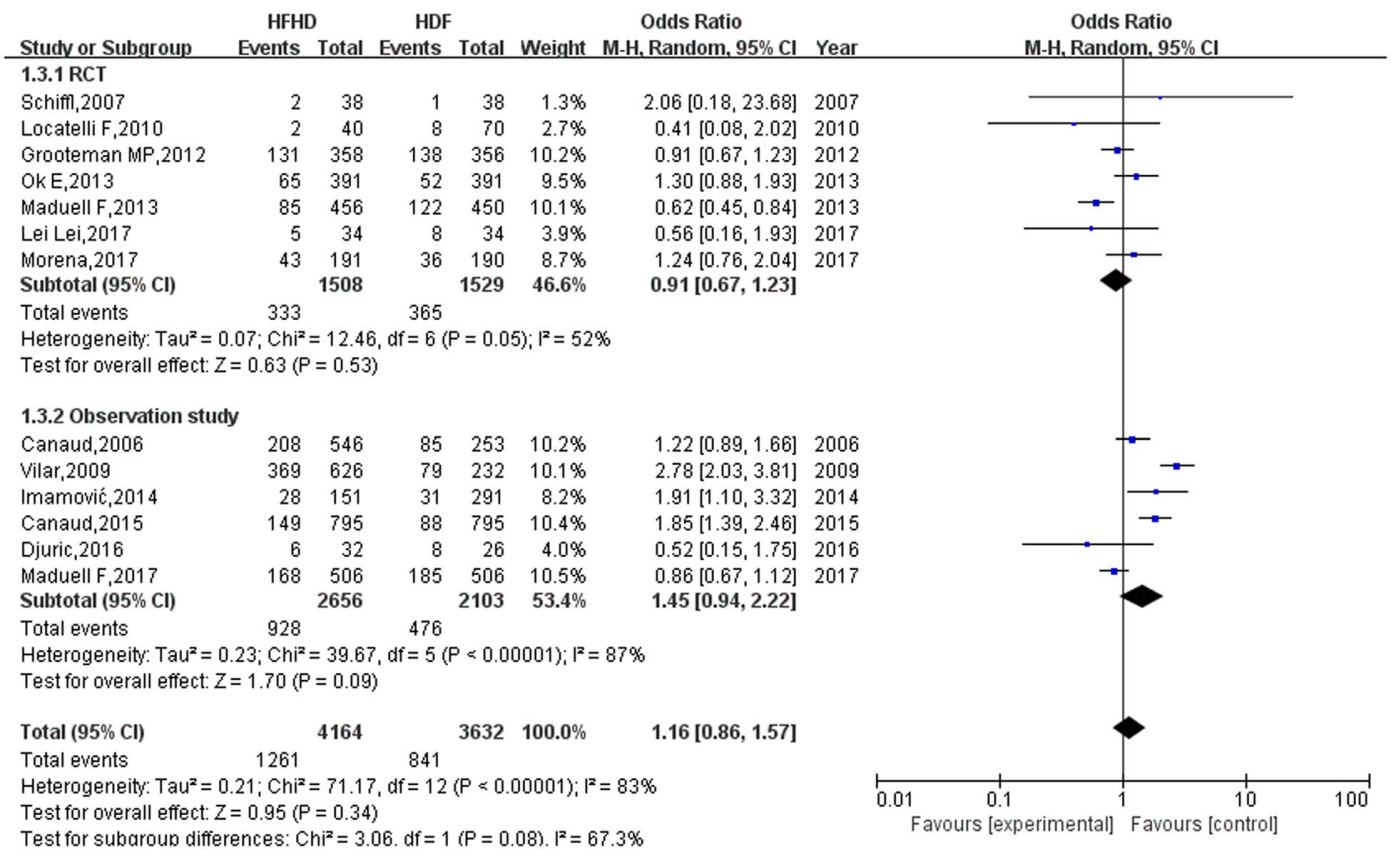
Subgroup analysis of all-cause mortality.

**Figure 8.**
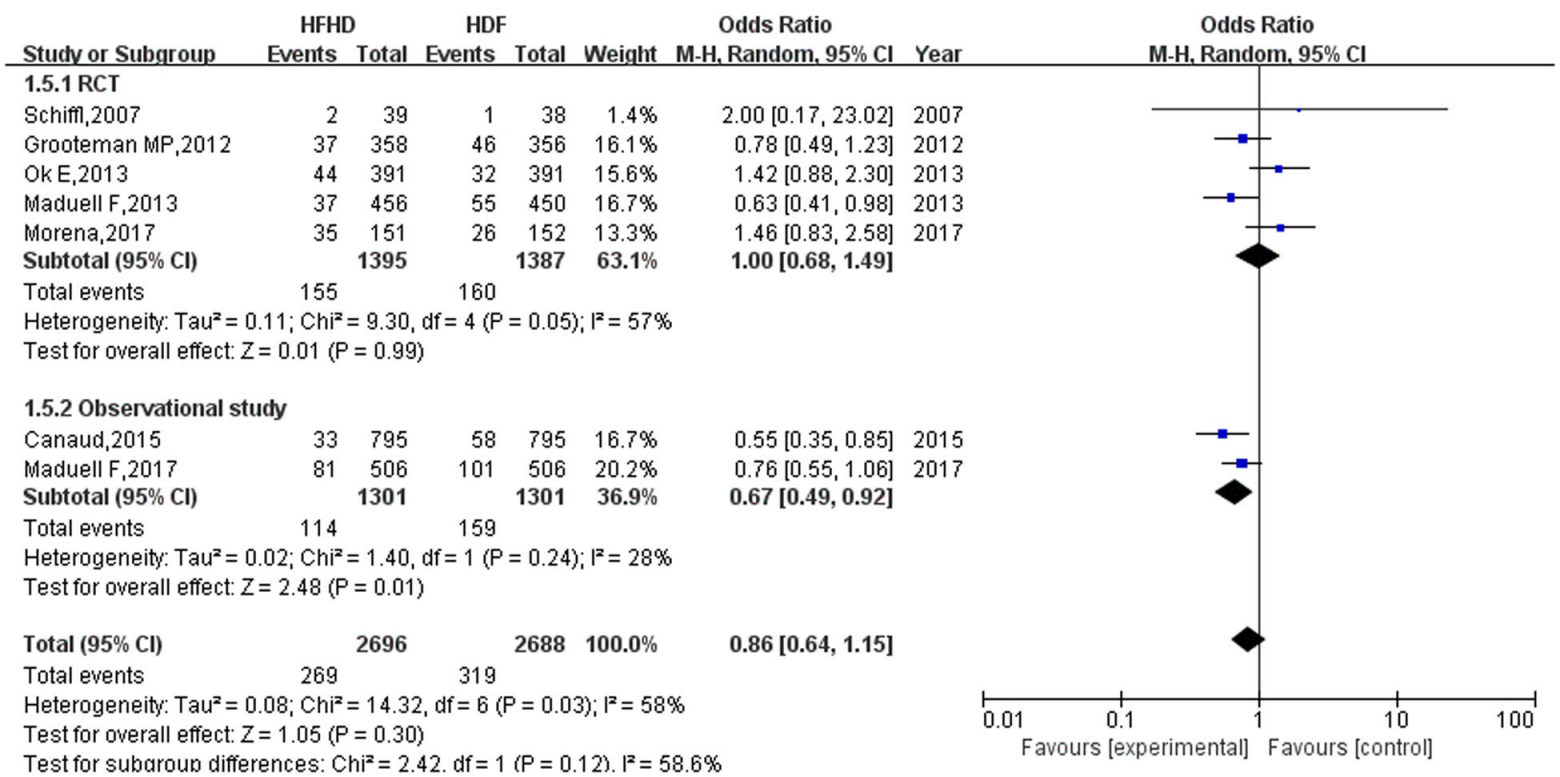
Subgroup analysis of cardiovascular mortality.

As randomized controlled trials have the strongest evidential profile in evidence-based research, we conducted subgroup analyses according to study type. For all-cause mortality, the subgroup analysis results found no obvious difference between randomized controlled trials and observational studies (Figure 7). However, the results on cardiovascular mortality demonstrated a difference between randomized controlled trials and observational studies. Although the randomized controlled trial results found no statistically significant difference between HFHD and HDF in terms of their effect on cardiovascular mortality (OR: 1.00, 95% CI: 0.68, 1.49, *p* = 0.99), the results of observational studies showed that HFHD reduced cardiovascular mortality (OR: 0.86, 95% CI: 0.64, 1.15, *p* = 0.01) (Figure 8).

### Publication bias

**Figure 9.**
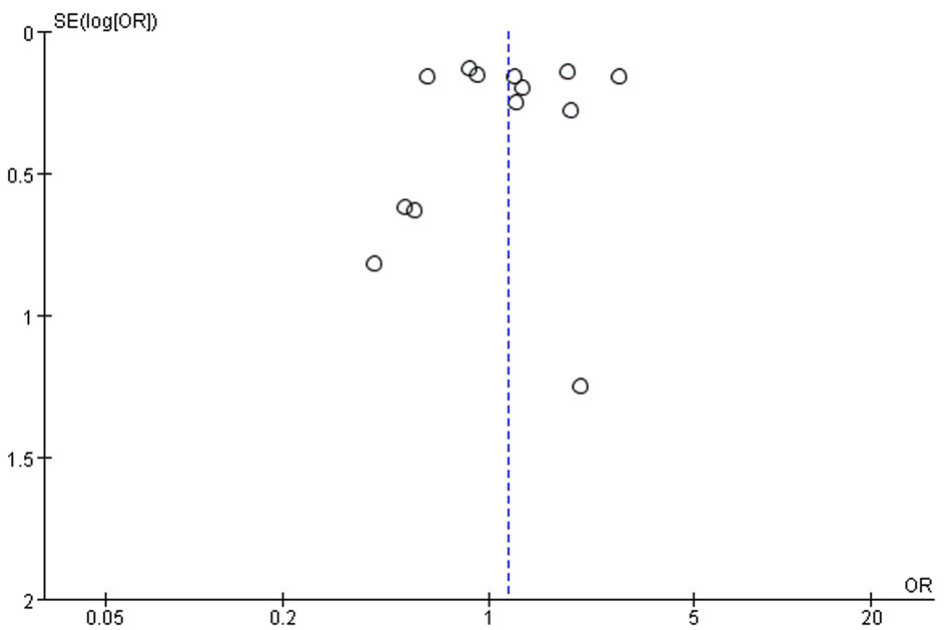
Funnel plot of all-cause mortality.

Since the 13 kinds of literature included in the study all reported all-cause mortality, we chose all-cause mortality as the indicator to evaluate publication bias. The funnel plot (Figure 9) results show that the funnel plot scatters are mainly concentrated at the top, and the distribution of scatters on both sides is symmetrical. This suggests that there is no obvious publication bias in the target literature of this study.

## Discussion

After a systematic search and screening of the literature, 13 studies were finally included in the meta-analysis of the effect of HFHD and HDF on the survival rate of patients with ESKD, involving 7796 patients with ESKD. Compared with the HDF mode, the HFHD mode significantly decreased infection mortality in patients with ESKD. The infection mortality rate in the HFHD group was 0.50 times lower than that in the HDF group (95% CI: 0.33, 0.77, *p* = 0.002). There was no significant difference between HFHD and HDF regarding all-cause mortality (OR: 1.16, 95% CI: 0.86, 1.57) or cardiovascular disease mortality in patients with ESKD (OR: 0.86; 95% CI: 0.64, 1.15, *p* = 0.30). However, a sensitivity analysis revealed that HFHD may have a positive effect on cardiovascular disease mortality (OR: 0.75, 95% CI: 0.62, 0.91). Additionally, to our knowledge, our study is the most comprehensive study including both randomized controlled trials and cohort studies to explore the effect of HFHD and HDF on the survival rate of patients with ESKD.

Previous studies have revealed that HDF reduces the all-cause mortality [25–27] and cardiovascular mortality [27,28] of patients with ESKD compared with conventional hemodialysis, suggesting that HFD has a protective effect on patients. In our study, although there was no statistically significant difference between HFHD and HDF, a meta-analysis that only included randomized controlled trials demonstrated that HFHD had a positive effect on reducing all-cause mortality and cardiovascular death [29]. In addition, Zhu [30] found that HFHD reduced infection mortality, which is consistent with our results. Most patients with ESKD retain part of their kidney function, which would promote phosphorus and reduce mortality [31,32]. However, kidney function gradually deteriorates during the dialysis process, and dialysis may have a large impact on the decline of the patients’ kidney function [33]. A meta-analysis containing randomized controlled trials and cohort studies indicated that HFHD and HFD may better protect residual kidney function measured by the endogenous creatinine clearance rate and urea clearance rate or urine volume compared with LFHD [34]. Additionally, some studies have indicated that the clearance rate of most toxic substances during HFHD is significantly higher than that during HFD, which can improve the kidney function of patients by reducing the accumulation of toxic substances in the blood [35,36].

It has been suggested that convective flow may affect mortality. Canaud et al. [14] performed propensity score matching in a cohort of 2293 patients and found that the relative survival improvement in OLHDF was related to convective flow. This means that when the flow rate reached 56.8 L per week, the survival rate increased linearly, and when the flow rate reached 75.0 L, the survival benefits were minimal. However, in patients undergoing maintenance hemodialysis therapy, achieving a high convective flow is influenced by factors such as vascular access, blood flow, blood viscosity, and the dialyzer [37,38]. Therefore, we can speculate that these influences may play a role in mortality. However, our study cannot answer this question at this time.

This study has the following limitations: (1) most of the studies included focused on European populations and therefore do not represent all races; (2) this meta-analysis was not registered online and did not include recent studies due to insufficient data; (3) in the included studies, the sample size of the three studies was small, less than 50, which may cause the results to be unrepresentative; (4) as the data included in the study were limited, we pooled the data of cohort studies and randomized controlled trials; and (5) most patients using HDF will choose kidney transplantation, and the censored data related to this group underestimated the role of HDF to some extent.

## Conclusions

In summary, compared with HDF, HFHD reduces infection mortality. Whereas HFHD may have a positive effect on cardiovascular disease mortality. Moreover, these modes have no statistically significant effect on all-cause mortality or cardiovascular death. The results of this study suggest that HDF is the preferred model for hemodialysis in patients with ESKD disease when considering infection mortality. However, due to the limitations of this study, further large-scale long-term multi-ethnic and multi-center cohort studies or randomized controlled trials are needed to verify the conclusions.

## Supplementary Material

Supplemental Material

## Data Availability

All data generated or analyzed during this study are included in this published article.
