# Supplementary material for: Effects of high-flux hemodialysis and hemodiafiltration on the mortality of patients with end-stage kidney disease: a meta-analysis
Source: Ren Fail. 2023 Jul 10;45(1):2147436. doi: 10.1080/0886022X.2022.2147436 (PMC10334859; doi:10.1080/0886022X.2022.2147436)
Supplement: Supplemental Material [file IRNF_A_2147436_SM0961.pdf]

Retrieval strategy

In line with the Preferred Reporting Items for Systematic Reviews and Meta-Analyses guidance manual, three English databases, PubMed, EMBASE and Cochrane Library, and three Chinese databases, CNKI, Wanfang and VIP, were systematically searched. The search period was up to 31 July 2022. English database retrieval strategies included the following keywords: “end-stage kidney disease”, “hemodiafiltration”, “high-flux hemodialysis”. Chinese database retrieval strategies include the same Chinese keywords. The details of the search strategy displayed as follow: (("kidney failure, chronic"[MeSH Terms] OR ("kidney"[All Fields] AND "failure"[All Fields] AND "chronic"[All Fields]) OR "chronic kidney failure"[All Fields] OR ("end"[All Fields] AND "stage"[All Fields] AND "kidney"[All Fields] AND "disease"[All Fields]) OR "end stage kidney disease"[All Fields]) AND ("haemodiafiltration"[All Fields] OR "hemodiafiltration"[MeSH Terms] OR "hemodiafiltration"[All Fields]) AND ("high-flux"[All Fields] AND ("haemodialysis"[All Fields] OR "renal dialysis"[MeSH Terms] OR ("renal"[All Fields] AND "dialysis"[All Fields]) OR "renal dialysis"[All Fields] OR "hemodialysis"[All Fields]))) NOT (("kidney failure, chronic"[MeSH Terms] OR ("kidney"[All Fields] AND "failure"[All Fields] AND "chronic"[All Fields]) OR "chronic kidney failure"[All Fields] OR ("end"[All Fields] AND "stage"[All Fields] AND "kidney"[All Fields] AND "disease"[All Fields]) OR "end stage kidney disease"[All Fields]) AND ("haemodiafiltration"[All Fields] OR "hemodiafiltration"[MeSH Terms] OR "hemodiafiltration"[All Fields]) AND

Information Classification: General

("high-flux"[All Fields] AND ("haemodialysis"[All Fields] OR "renal dialysis"[MeSH Terms] OR ("renal"[All Fields] AND "dialysis"[All Fields]) OR "renal dialysis"[All Fields] OR "hemodialysis"[All Fields])) AND "animals"[MeSH Terms:noexp])

Information Classification: General
